# Statistical modeling for temporal dominance of sensations data incorporating individual characteristics of panelists: an application to data of milk chocolate

**DOI:** 10.1007/s13197-021-05260-9

**Published:** 2021-09-24

**Authors:** Sumito Kurata, Reiko Kuroda, Fumiyasu Komaki

**Affiliations:** 1 Graduate School of Information Science and Technology, The University of Tokyo, 7-3-1 Hongo, Bunkyo-ku, Tokyo, 113-8656 Japan; 2 LOTTE Co., Ltd. Central Laboratory, 1-1 Numakage 3-chome, Minami-ku, Saitama, 336-8601 Japan; 3 RIKEN Center for Brain Science, 2-1 Hirosawa, Wako City, Saitama, 351-0198 Japan

**Keywords:** Temporal dominance of sensations (TDS), Chocolate, Semi-Markov chain, Generalized linear model, Negative binomial regression

## Abstract

We discuss the modeling of temporal dominance of sensations (TDS) data, time series data appearing in sensory analysis, that describe temporal changes of the dominant taste in the oral cavity. Our aims were to obtain the transition process of attributes (tastes and mouthfeels) in the oral cavity, to express the tendency of dominance durations of attributes, and to specify factors (such as sex, age, food preference, dietary habits, and sensitivity to a particular taste) affecting dominance durations, simultaneously. To achieve these aims, we propose an analysis procedure applying models based on the semi-Markov chain and the negative binomial regression, one of the generalized linear models. By using our method, we can take differences among individual panelists and dominant attributes into account. We analyzed TDS data for milk chocolate with the proposed method and verified the performance of our model compared with conventional analysis methods. We found that our proposed model outperformed conventional ones; moreover, we identified factors that have effects on dominance durations. Results of an experiment support the importance of reflecting characteristics of panelists and attributes.

**Supplementary Information:**

The online version contains supplementary material available at 10.1007/s13197-021-05260-9.

## Introduction

Temporal dominance of sensations (TDS) data are the time series data used in sensory analysis and give information about temporal characteristics of a sample (food or drink), for example, “sweetness comes after bitterness” or “saltiness lasts long in the mouth” (Pineau et al. 2003). This series consists of changes in the dominant attribute (taste or mouthfeel) as reported by panelists (subjects). TDS experiments are generally conducted on a PC screen. After taking a sample into their mouth, a panelist feels many types of tastes and mouthfeels in their oral cavity. Each panelist reports the most striking perceptions from among a list of candidate attributes given in advance, at each time point. When a panelist feels a change of the dominating attribute, he or she clicks the corresponding button on the screen. If he or she no longer feels a dominant attribute, he or she finishes the experiment by clicking the finish button. In many cases, the length of observed TDS data is 1 or 2 minutes, and we obtain the data at intervals of 1 second. Since the time that a panelist requires to finish tasting depends on the person, the length of TDS data also varies by panelist.

To analyze TDS data, TDS curves have been used in many studies. TDS curve provides dominance rate of each attribute at each time, and thus average changing process of dominant attributes over panelists. However, we cannot reflect the characteristics of individual panelists in TDS curves, and it is difficult to integrate data among panelists adequately because time length of TDS data is not uniform among panelists. Recently, some studies applied statistical analysis methods to TDS data (e.g., Dinnella et al. 2013; Le Révérend et al. 2008; Okamoto et al. 2020). One representative line of research is the introduction of Markov models (e.g., Cardot et al. 2019; Franczak et al. 2015; Lecuelle et al. 2018). By using the Markov chain (MC: Markov 1971), we obtain transition probabilities that describe the probability to change from one state to another state in a single time unit. In TDS data analysis, a “state” corresponds to a dominant attribute. Hence, we can solve the problem of determining which attribute tends to change to which attribute by using MC. Moreover, some studies (e.g., Lecuelle et al. 2018) discussed durations of dominant attributes by introducing semi-Markov chains (SMC: Lévy 1954; Smith 1958). The concept of SMC expands the traditional MC in terms of probability distributions of sojourn times of states. In TDS data analysis, sojourn times correspond to dominance durations of attributes, and thus, we can discuss the tendencies of times for which attributes last in the oral cavity.

In this paper, we mainly focus on the modeling of dominance durations by applying generalized linear models (GLM: Nelder and Wedderburn 1972). Particularly, we consider how to reflect the characteristics of each panelist and dominant attribute. Note that most conventional studies to date have not taken characteristics of panelists (e.g., sex, age, and food preference) into account. However, it is more natural that some characteristics affect the dominance durations; for example, “males tend to feel dominant tastes longer than females” or “individuals who prefer sweet food tend to feel tastes longer”. Therefore, we employ many types of characteristics of panelists as explanatory variables in a regression model. We expand conventional models to reflect characteristics of panelists and dominant attributes by utilizing negative binomial regression (NBR), one of the GLM. NBR is a statistical modeling method to express the relationship between a response variable and explanatory variables based on the negative binomial distribution (NBD). Moreover, to express the differences of tendencies of dominance duration between groups, like “bitterness lasts longer than saltiness in the mouth of the young, but shorter in the mouth of the elderly”, we divide attributes into some groups in conformity with characteristics (e.g., “taste/mouthfeel” and “sweet/sour/salty/bitter/umami”). By assigning different values of parameters based on in which group each attribute belongs, we can express differences among attribute groups. Our major aim was to obtain not only the transition process but also characteristics that have effects on dominance durations.

## Material and methods

### Product

In this paper, we show real data analysis using milk chocolate with greater than 21 percent cocoa, obtained from LOTTE Co., Ltd. (Tokyo, Japan). Ingredients included in the milk chocolate are as follows: Sugar, whole milk powder, cacao mass, cocoa butter, vegetable oils, emulsifier (soy origin), vegetable lecithin, and flavorings. The objective of this experiment was to express the transition of tastes of this chocolate in the oral cavity and to identify which factor has effects on dominance durations, by utilizing a Markov model and NBR.

### Sensory evaluation

We conducted TDS experiment to analyze the material. The number of panelists was 54 (29 male and 25 female). The youngest and oldest panelists were 24 and 64 years old, respectively. After a discussion with experts at LOTTE Co., Ltd., we chose to employ the 9 dominant attributes listed in Table 1. Note that, “Cacao” (A1) and “Cocoa” (A3) differ in terms of bitterness/sweetness: A1 means bitter taste and aroma derived from cacao mass, and A3 indicates sweetness derived from cocoa. Then, from the viewpoint of properties, we divided the attributes into 4 groups: Group I and Group II consist of attributes related to bitterness (“Cacao” and “Roast”) and sweetness (“Cocoa”, “Milk”, “Vanilla”, and “Caramel”), respectively; Group III is composed of attributes related to mouthfeel (“Nutty” and “Cohesiveness”); and Group IV has only “Richness” as its element. Average and standard deviation of time lengths of TDS data were 81.0 and 23.7 seconds, respectively. We show the histogram of the observed 367 dominance durations in Fig. S1 in Online Resource.

**Table 1:** List of dominant attributes used in our experiment and attribute groups

Index	Attribute	Group
A1	Cacao	I
A2	Roast	I
A3	Cocoa	II
A4	Milk	II
A5	Vanilla	II
A6	Caramel	II
A7	Nutty	III
A8	Cohesiveness	III
A9	Richness	IV

### Statistical methods

#### Temporal modeling based on semi-Markov chains

Let *I* and *J* be the numbers of panelists and dominant attributes. In our experiment, $I=54$ and $J=9$. We define the number of observed dominance durations of the *j*-th attribute answered by the *i*-th panelist as $C_{i, j}$ ($i \in \{ 1 \,,\,\ldots \,,\, I \}$, $j \in \{ 1 \,,\,\ldots \,,\, J \}$). $C_{i, j}$ is zero if the *i*-th panelist never felt the *j*-th attribute dominantly during the experiment. SMC represents a time series by transition probabilities and dominance durations. The transition probabilities, which provide probabilities to change from one attribute to another in a single time unit (one second), are calculated from the frequencies of changes among attributes. We will describe the detailed definition and likelihood of the model in the appendix.

**Fig. 1:**
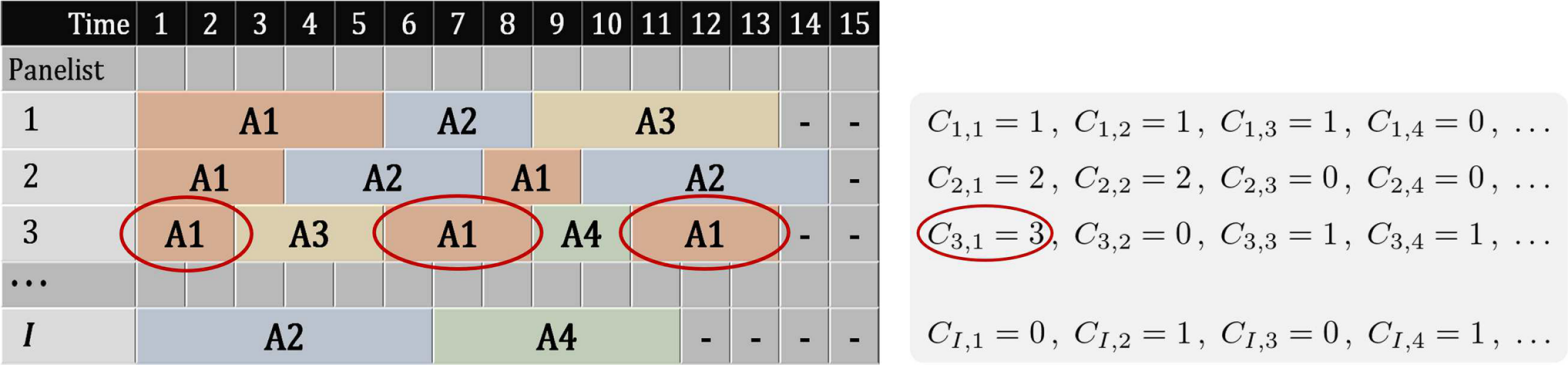
A synthetic TDS dataset and list of the values of $C_{i, j}$. Each row indicates the answers of a panelist. “-” means that the corresponding panelist has finished the experiment. For example, the 3rd panelist ($i=3$) felt attribute A1 ($j=1$) dominantly 3 times, i.e., $C_{3, 1} = 3$. Next, the observed 1st ($c=1$) dominance duration of A1 was 2 seconds, i.e., $Y_{3, 1, 1} = 2$. Similarly, we find $Y_{3, 1, 2} = 3$ and $Y_{3, 1, 3} = 3$. We can calculate $Y_{i, j, c}$ of other panelists in the same way

In this paper, we mainly focus on the modeling of dominance durations. Let $Y_{i, j, c}$ be the *c*-th observed duration of the *j*-th attribute obtained from the *i*-th panelist ($i \in \{ 1 \,,\,\ldots \,,\, I \}$, $j \in \{ 1 \,,\,\ldots \,,\, J \}$, $c \in \{ 1 \,,\,\ldots \,,\, C_{i, j} \}$). Note that, as a panelist can feel the same attribute dominantly more than once or never, the index set $\{ 1 \,,\,\ldots \,,\, C_{i, j} \}$ is a finite set or an empty set. We show a synthetic example of TDS data, and values of $C_{i, j}$ and $Y_{i, j, c}$ in Fig. 1.

Now, we define a probability that indicates the panelist feels an attribute dominantly for a certain number of seconds (*y*); for each *i*, *j*, *c*, and *y*,

1 $$\begin{aligned} f_{i, j}(y) \,=\, \mathbf {P} \bigl \{ Y_{i, j, c} \,=\, y \;{\big |}\; j \bigr \} \,. \end{aligned}$$

This can depend on two factors: Who is the corresponding panelist (*i*) and what is the dominating attribute (*j*). The traditional MC implicitly assumes that dominance durations follow a geometric distribution (GD), while SMC permits wide variety of probability distributions, such as the Poisson distribution (PD) and NBD. Some conventional studies reported that NBD is more suitable as the distribution of dominance durations than GD (e.g., Lecuelle et al. 2018).

#### Proposed method: reflecting characteristics of panelists and attributes by negative binomial regression models

Using SMC, we can conduct flexible modeling of dominance durations, but we implicitly assume that there is no effect of characteristics of panelists on the durations in simple applications. On the other hand, it is expected that some factors related to panelist characteristics and attributes also influence dominance durations. Hence, we now introduce a flexible model by utilizing NBR. NBR is suitable for cases where the response variable is non-negative integer data (e.g., Allison and Waterman 2002; Lawless 1987). Let $\varvec{x}_{i}$ $=$ $\left( x_{i, 1} \,,\,\ldots \,,\, x_{i, M} \right)$ be the *M*-dimensional covariate vector related to the *i*-th panelist, consisting of variables that can affect the dominance durations, such as sex, age, and food preference. In our setting, $\{ Y_{i, j, c} \}$ and $\{ \varvec{x}_{i} \}$ correspond to the response variables and explanatory variables, respectively. Note that, in our experiment, individual data that including missing values of explanatory variables were omitted, and each explanatory variable was normalized.

We next consider the grouping of attributes. We assume that there are *G* ($1 \le G \le J$) attribute groups, and attributes in the same attribute group have some kind of common characteristic, for example, “taste/mouthfeel” and “sweet/sour/salty/bitter/umami”. We divide the index set of attributes, $\{ 1 \,,\,\ldots \,,\, J \}$, into *G* subsets, $\mathcal {J}_{1} \,,\,\ldots \,,\, \mathcal {J}_{G}$. We can express characteristics of each attribute group by assigning parameters different values according to in which group the corresponding attribute belongs.

We now assume that, for each $i \in \{ 1 \,,\,\ldots \,,\, I \}$ and $j \in \{ 1 \,,\,\ldots \,,\, J \}$, $Y_{i, j, c}$ ($c \in \{ 1 \,,\,\ldots \,,\, C_{i, j} \}$) independently follows the NBD whose probability function is defined as follows:

2 $$\begin{aligned}&f_{i, j}^{\mathrm {NBR}}(Y_{i, j, c}) \,=\, \frac{\Gamma (Y_{i, j, c}+\tilde{\kappa }_{j})}{\Gamma (Y_{i, j, c}+1) \, \Gamma (\tilde{\kappa }_{j})} \, \left( \frac{\mu _{i, j}}{\mu _{i, j} + \tilde{\kappa }_{j}} \right) ^{Y_{i, j, c}} \left( \frac{\tilde{\kappa }_{j}}{\mu _{i, j} + \tilde{\kappa }_{j}} \right) ^{\tilde{\kappa }_{j}} \,, \\&\mu _{i, j} \,=\, \mu (\varvec{x}_{i}\,;\,\tilde{\varvec{\beta }}_{j}) \,=\, \exp \left( \tilde{\varvec{\beta }}_{j}^{\mathrm {T}} \varvec{x}_{i} \right) \,, \nonumber \end{aligned}$$

where $\tilde{\varvec{\beta }}_{j}^{\mathrm {T}}$ is the transpose of the *M*-dimensional regression coefficient vector $\tilde{\varvec{\beta }}_{j}$. The values of regression coefficient vector $\tilde{\varvec{\beta }}_{j}$ and parameter $\tilde{\kappa }_{j}$ vary depending on the attribute group:

$$\begin{aligned} & \tilde{\varvec{\beta }}_{j} \,=\, \left\{ \begin{array}{ll} \varvec{\beta }_{1} \,=\, \left( \beta _{1, 0} \,,\, \beta _{1, 1} \,,\,\ldots \,,\, \beta _{1, M} \right) &{} \left( j \,\in \, \mathcal {J}_{1} \right) \\ \;\vdots &{} \\ \varvec{\beta }_{G} \,=\, \left( \beta _{G, 0} \,,\, \beta _{G, 1} \,,\,\ldots \,,\, \beta _{G, M} \right) &{} \left( j \,\in \, \mathcal {J}_{G} \right) \\ \end{array} \right. \, , \\ & \tilde{\kappa }_{j} \,=\, \left\{ \begin{array}{ll} \kappa _{1} &{} \left( j \,\in \, \mathcal {J}_{1} \right) \\ \;\vdots &{} \\ \kappa _{G} &{} \left( j \,\in \, \mathcal {J}_{G} \right) \\ \end{array} \right. \,, \end{aligned}$$

where $\beta _{g, 0}$ $(g \in \{ 1 \,,\,\ldots \,,\, G \})$ is the intercept term of the *g*-th attribute group. Hereafter, we use the notation $Y_{i, j, c} \sim \mathrm {NBR}\left( \mu _{i, j} \,,\, \tilde{\kappa }_{j} \right)$ if random variable $Y_{i, j, c}$ has Eq. (2) as its probability function. The expectation of this probability distribution is $\mu _{i, j}$ and depends on the linear combination of explanatory variables. This model can express which explanatory variable (characteristic of panelists) has an effect to lengthen or shorten dominance durations, for example, “the young tends to feel attributes shorter than the elderly”. If $\beta _{g, m}$ is positive for a certain $g \in \{ 1 \,,\,\ldots \,,\, G \}$ and $m \in \{ 1 \,,\,\ldots \,,\, M \}$, the expectation of the distribution becomes larger as the value of the corresponding explanatory variable becomes larger, and thus, the *m*-th explanatory variable has an effect to lengthen the dominance durations of attributes in the *g*-th attribute group. Conversely, we can consider that the *m*-th explanatory variable tends to shorten the dominance durations of the *g*-th attribute group if $\beta _{g, m} < 0$. Additionally, parameters $\kappa _{1} \,,\,\ldots \,,\, \kappa _{G}$ $(> 0)$ play roles in shaping the distribution without changing the expectation, and thus we can express the different tendencies of dominance durations between attribute groups by $\kappa _{1} \,,\,\ldots \,,\, \kappa _{G}$. Hereafter, we refer to the $\kappa$’s as shape parameters.

We estimate regression coefficients and shape parameters by maximizing a likelihood function (see also the "appendix"). Remark that, it is not desirable to subdivide parameters beyond necessity, because dividing increases the number of parameters, and a model that employs too many parameters often falls into over-fitting. In the case of NBR models, when we divide parameters according to *G* attribute groups, we need *G* times the number of parameters of the model without divisions. Generally, to estimate a large number of parameters stably, an even larger sample size is needed; that is to say, this approach needs quite a large number of panelists and long time lengths of TDS data. On the other hand, the number of panelists is at most a few dozen and the time length of TDS data is 1 or 2 minutes, in many cases. Hence, in our experiment, we did not divide parameters into 9 attributes but into $G=4$ attribute groups (Groups I– IV) to avoid over-fitting.

#### Statistical models used in our experiment

To express dominance durations, we used the following models.

*[G: Model based on GD]*

Model G coincides with a traditional MC. As mentioned in the subsection “Statistical methods”, MC assumes substantially that dominance durations have GD. The required number of parameters in this model is 1.

*[N and Ng: Models based on NBD]*

Model N is a SMC employing NBD as the probability distribution of dominance durations. Note that, since Model N (and Model G) assume that values of parameters are common across attributes, these models cannot reflect differences of effects among attributes. Model Ng is an expansion of Model N; it divides parameters in NBD according to *G* attribute groups. Regarding the parameters employed in GD and NBD, these will be described in the "appendix". The required numbers of parameters in Model N and Model Ng are 2 and 2*G*, respectively.

*[R and Rg: Models based on NBR (proposed methods)]*

Model R and Model Rg are SMC utilizing NBR. Model Rg is an expansion of Model R; it divides the regression coefficient and shape parameter according to *G* attribute groups. When we employ *M* explanatory variables, the required numbers of parameters including intercept and shape parameters in Model R and Model Rg are $M+2$ and $(M+2)G$, respectively. We can prove that the NBR models include the regression models based on GD and PD, and some of the conventional models using SMC are special cases of NBR models (for details, see the "appendix"). Consequently, Model Rg includes the other models used in our experiment.

#### On explanatory variables

We prepared 11 candidate covariates that can have effects on way of feeling of tastes. The candidates of explanatory variables employed in the NBR models (Model R and Model Rg) are listed in Table 2. Values of “Food preference” (V05, V06, V07) were on a scale of 1 (very unpreferable) to 5 (very preferable). Values of “Taste recognition threshold” (V09, V10, V11) are obtained from results of threshold tests of sensitivity to sweetness, saltiness, and bitterness, respectively. The threshold test measures the limit of concentration at which a panelist can detect the object corresponding to a certain taste in a water solution (for details, see Meilgaard 1991). Following conventional research (Furukawa and Ueda 2012), we used granulated sugar (sweetness), table salt (saltiness), and anhydrous caffeine (bitterness) as the objects to be detected, and we set five levels of concentration. For each panelist, the value of “Taste recognition threshold” is the weakest concentration at which he or she detected the presence of the object in the water solution. Namely, the smaller the value of “Taste recognition threshold” for a taste is, the more sensitive to the corresponding taste he or she is.

**Table 2:** List of explanatory variables employed in our experiment

Index	Explanatory variable
V01	Sex (male=1, female=0)
V02	Age
V03	Smoking history (yes=1, no=0)
V04	Preference for fatty food (yes=1, no=0)
V05	Food preference (sweetness)
V06	Food preference (saltiness)
V07	Food preference (bitterness)
V08	Number of homemade dishes eaten per week
V09	Taste recognition threshold (sweetness)
V10	Taste recognition threshold (saltiness)
V11	Taste recognition threshold (bitterness)

Since not all of the explanatory variables affect dominance durations, for the present study, we selected optimal variables by the Akaike information criterion (AIC: Akaike 1974). AIC is a measure of the farness of a model from the true distribution (data-generating structure). The definition is as follows:

$$\begin{aligned} \mathrm {AIC} \,=\, & -2 \times (\mathrm {maximum \, log \, likelihood}) \\ & \; + 2 \times (\mathrm {the \, number \, of \, parameters}) \,. \end{aligned}$$

The form of likelihood of our proposed model will be described in the "appendix". Remark that the most complex regression model (employing all candidates of explanatory variables) would definitely be selected if we compared models by likelihood, whereas we can take the redundancy of a variable subset into account by utilizing AIC.

## Results and discussion

Here we show the estimated results of each model and discuss the effects of characteristics of panelists and dominant attributes. We conducted data analysis by using R, a software environment for statistical computing and graphics (R Core 2020).

**Fig. 2:**
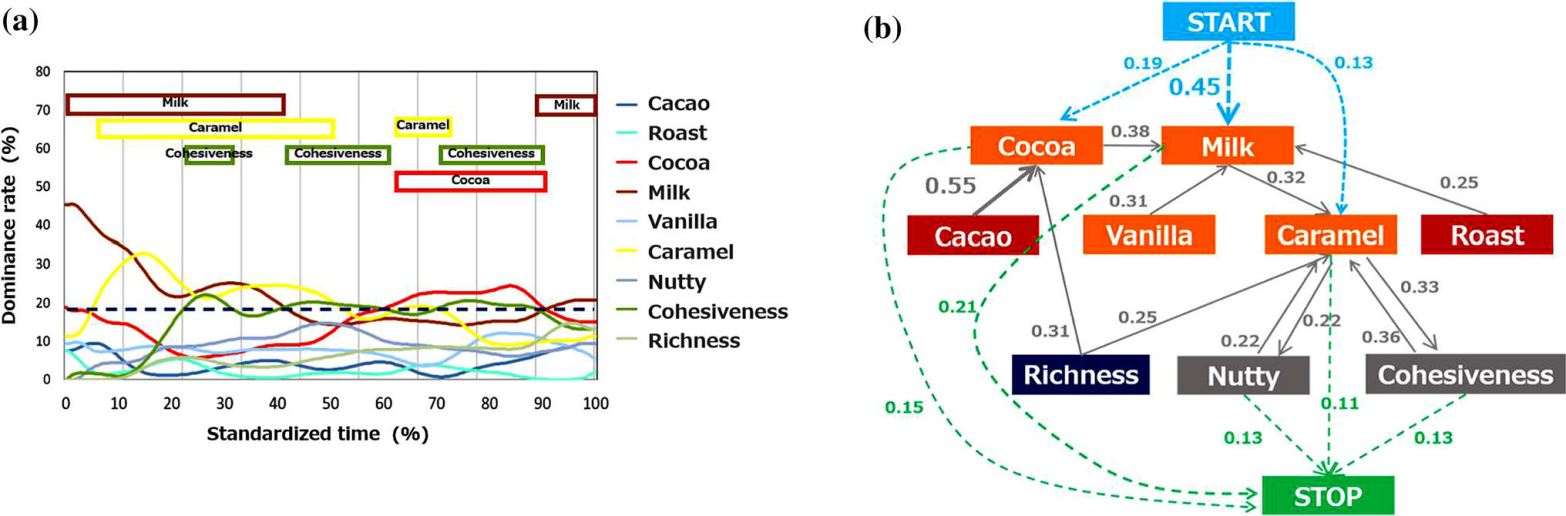
(a) TDS curves obtained in our experiment. The vertical and horizontal axes indicate dominance rate of each attribute and standardized time, respectively. The dotted line means the significance level (5%). The upper bands indicate attributes whose dominance rates exceeded the significance level during the corresponding period;  (b) Estimated transition probabilities of SMC. Each numerical value with an arrow is a transition probability. Transition probabilities less than 0.20 are omitted. Numerical values with arrows from “START” are estimated initial probabilities, and values with arrows pointing to “STOP” are the relative frequencies of transitions from an attribute to the end of experiment. Values less than 0.10 are omitted

We first show the obtained TDS curve and estimated transition probabilities in Fig. 2. From the standardized TDS curve (Fig. 2a), we find that almost half of the panelists felt “Milk” firstly. “Caramel” also recorded a high dominance rate in the first half of the tasting period. During the latter half, the dominance rate of “Cocoa” increased. “Cocoa”, “Milk”, and “Caramel” in Group II, and “Cohesiveness” in Group III exceeded the 5% significance level (dotted line) during the experiment. Additionally, from the estimated transition probabilities (Fig. 2b), we can see the changing process of taste and mouthfeel in the oral cavity; for example, “Cocoa” tends to come from “Cacao”, which changes to “Milk”. Remark that these are interpretations of average values, and thus the curve does not consider the differences among individuals.

The AIC values of Models G, N, Ng, R, and Rg were 2490.83, 2436.36, 2427.01, 2404.48, and 2396.30, respectively. We confirm that our proposed methods (Model R and Model Rg) outperformed the conventional ones, and Model Rg recorded the best (smallest) AIC.

### Results of model R

Table 3 gives the values of regression coefficients and the shape parameter estimated by Model R. As a result of model selection by AIC, the $M=5$ (of 11) explanatory variables shown in Table 3 were selected. This model outperformed the conventional methods (Model G, Model N, and Model Ng) in terms of AIC, and thus we can see the importance of characteristics of panelists. The estimated results suggest the following: Males feel the same dominant attributes longer than females (V01); individuals who prefer fatty, sweet, and not-salty foods feel the same dominant attributes longer (V04, V05, V06); individuals who are sensitive to saltiness feel the same dominant attributes shorter (V10).

**Table 3:** Estimated values of regression coefficients and shape parameter (denoted by $\kappa$) in Model R

Attribute	Intercept	V01	V04	V05	V06	V10	$\kappa$
All	2.362	0.082	0.141	0.135	$-0.111$	$-0.129$	2.097

### Results of model Rg

Table 4 gives the values of the regression coefficients and the shape parameter of each attribute group as estimated by Model Rg. The $M=8$ (of 11) explanatory variables were selected by AIC. This model can express differences of tendencies between the attribute groups as well as characteristics of panelists. Some of the explanatory variables not employed in Model R were used in Model Rg; this is regarded as an effect of dividing parameters according to the attribute groups. This model recorded the best AIC in our experiment.

**Table 4:** Estimated values of regression coefficients and shape parameter (denoted by $\kappa$) of each attribute group in Model Rg

Attribute group	Intercept	V01	V02	V04	V05	V06	V08	V10	V11	$\kappa$
I	1.35	$-0.07$	0.33	0.21	0.10	$-0.26$	$-0.31$	0.38	$-1.41$	70.31
II	2.36	0.15	$-0.07$	0.22	0.10	$-0.08$	0.11	$-0.13$	0.03	2.04
III	2.35	$-0.09$	$-0.01$	$-0.02$	0.29	$-0.18$	$-0.08$	$-0.21$	0.08	2.58
IV	2.53	0.18	0.30	0.23	$-0.20$	0.00	$-0.31$	0.15	$-0.04$	21.55

The estimated results suggest the existence of some relationships between characteristics of the panelists and the attribute groups. A notable example is that a larger value of the taste recognition threshold for bitterness (V11) markedly shortens the dominance durations of attributes in Group I (consisting of attributes related to bitter taste), while it has little effect on other attribute groups. In other words, a panelist who is sensitive to bitterness is prone to detecting bitterness. It has been suggested that individuals who do not eat much bitter food tend to be sensitive to bitterness (e.g., Tanimura and Mattes 1993). It is thought that bitter tastes play the role of alerting the eater to the existence of toxicity (e.g., Fischer et al. 2005). Although milk chocolate is normally categorized as a sweet food and it is seldom recognized as a toxic compound, it is possible that individuals who are sensitive to bitterness can detect a small amount of bitterness in a contained compound acutely. Moreover, we can confirm differences of tendencies due to sex and age. From Table 4, we find that males tend to feel sweetness (corresponding to Group II) and richness (Group IV) longer relative to females, while bitterness (Group I) and mouthfeel (Group III) are felt shorter. Also, we see that the elderly tend to feel bitterness and richness longer relative to the young, but sweetness is felt shorter. In many studies, differences of preference and sensitivity (as reflected in detection and recognition thresholds) due to sex and age have been pointed out (e.g., Mitsuhashi et al. 2008; Narukawa and Misaka 2020; Spence 2019). Our results suggest that sex differences and age differences have some effects on not only food preference and sensitivity of taste but also dominance duration in the oral cavity.

## Conclusions

In this paper, we introduced statistical models for analyzing TDS data and applied them to real data for milk chocolate. Our methods build upon SMC and NBR, in order to reflect characteristics of panelists and differences among dominant attributes. Using the proposed model, we can obtain transition process of attributes, tendency of dominance durations, and factors that have effects on dominance durations, simultaneously. By dividing parameters according to attribute groups appropriately, we clarify correspondence between personal characteristics and tastes. In real data analysis using milk chocolate, the proposed models outperformed conventional ones in terms of fitting. Our results support that sex, age, food preference, and dietary habits have effects on feelings of taste and mouthfeel, as much as sensitivity to a particular taste. In assigning panelists, prior knowledge about products and sensitivity to tastes (for example, in terms of the taste recognition threshold) are generally valued, but it has been suggested that reliability is improved by taking other factors such as those employed in our experiment into account.

Incidentally, tendencies of dominant attributes in the oral cavity may depend on time. To express time-varying characteristics, splitting TDS data into multiple time periods should be considered, as in several conventional studies (e.g., Dinnella et al. 2013; Kawasaki et al. 2019). Since change points of the time series can vary between individuals, it is further desirable to determine the change points for each panelist automatically. Additionally, improvement of modeling of transitions in SMC is another future task. SMC represents a time series by sojourn times and transition probabilities. As with the case of sojourn times (dominance durations), we expect to conduct flexible modeling with regard to transitions by employing information about panelists and attributes in future studies.

### Supplementary Information

Below is the link to the electronic supplementary material.

Supplementary file1 (PDF 413 kb)

## Data Availability

The datasets generated and analysed during the current study are not publicly available due to business reasons, but are available from the corresponding author on reasonable request except personal information.

## A Details of statistical models

### A.1 Markov chain

The (traditional) MC is a stochastic model that describes a sequence of states, corresponding to the dominant attributes. Let *I* and *J* be the numbers of panelists and attributes. Further, let $Z_{i}(t)$ be the dominant attribute at discrete time point *t* $( \in \{ 1 ,\, 2 ,\, \ldots \})$ for the *i*-th panelist ($i \in \{ 1 \,,\,\ldots \,,\, I \}$), and let $T_{i}$ be the length of the time series obtained from the *i*-th panelist. MC assumes the Markov property, that the conditional probability of being at the current dominant attribute depends only on the previous one:

$$ \begin{aligned} \mathbf {P} \bigl \{ Z_{i}(t) \,{\big |}\, Z_{i}(1) \,,\,\ldots \,,\, Z_{i}(t-1) \bigr \} \,=\, \mathbf {P} \bigl \{ Z_{i}(t) \,{\big |}\, Z_{i}(t-1) \bigr \} \quad \left( t \,\ge \, 2 \right) \,, \end{aligned} $$

for each $i \in \{ 1 \,,\,\ldots \,,\, I \}$. MC outputs a transition probability matrix, which is a stochastic matrix consisting of $P_{j, j'}$, the transition probabilities of each pair of attributes:

$$ \begin{aligned} P_{j, j'} \,=\, \mathbf {P} \bigl \{ Z_{i}(t) \,=\, j' \,{\big |}\, Z_{i}(t-1) \,=\, j \bigr \} \quad \bigl ( j ,\, j' \,\in \, \{ 1 \,,\,\ldots \,,\, J \} \bigr ) \,. \end{aligned} $$

Each transition probability is calculated as $m_{j, j'} / m_{j, \cdot }$, where $m_{j, j'}$ and $m_{j, \cdot }$ are the total numbers of transitions from the *j*-th attribute to the $j'$-th attribute and transitions from the *j*-th attribute to any attribute except itself, respectively. Additionally, let $\delta _{j}$ be the initial probability of the *j*-th attribute ($j \in \{ 1 \,,\,\ldots \,,\, J \}$); i.e., $\delta _{j} = \mathbf {P} \left\{ Z_{i}(1) \,=\, j \right\}$ for each *j*.

### A.2 Semi-Markov chain

SMC is an expansion of the traditional MC specifically for the probability distributions of sojourn times (corresponding to dominance durations). We define two sets, $\underline{\mathcal {C}}_{i, j}$ and $\overline{\mathcal {C}}_{i, j}$, for each $i \in \{ 1 \,,\,\ldots \,,\, I \}$ and $j \in \{ 1 \,,\,\ldots \,,\, J \}$ as follows:

$$ \begin{aligned}&\underline{\mathcal {C}}_{i, j} \,=\, \left\{ \begin{array}{ll} \left\{ 1 \right\} \,\cup \, \bigl \{ 2 \le t \le T_{i} \;{\big |}\; Z_{i}(t-1) \ne Z_{i}(t) = j \bigr \} &{} \quad \left( Z_{i}(1) \,=\, j \right) \\ \bigl \{ 2 \le t \le T_{i} \;{\big |}\; Z_{i}(t-1) \ne Z_{i}(t) = j \bigr \} &{} \quad (\mathrm {otherwise}) \\ \end{array} \right. \,, \\&\overline{\mathcal {C}}_{i, j} \,=\, \left\{ \begin{array}{ll} \bigl \{ 2 \le t \le T_{i} \;{\big |}\; Z_{i}(t) \ne Z_{i}(t-1) = j \bigr \} \,\cup \, \left\{ T_{i}+1 \right\} &{} \quad \left( Z_{i}(T_{i}) \,=\, j \right) \\ \bigl \{ 2 \le t \le T_{i} \;{\big |}\; Z_{i}(t) \ne Z_{i}(t-1) = j \bigr \} &{} \quad (\mathrm {otherwise}) \\ \end{array} \right. \,. \end{aligned} $$

These sets consist of the time points at which a change from an attribute not *j* to *j* and a change from *j* to another attribute occur, respectively. The numbers of elements of the two sets $\underline{\mathcal {C}}_{i, j}$ and $\overline{\mathcal {C}}_{i, j}$ are the same (they coincide with $C_{i, j}$). We represent elements of $\underline{\mathcal {C}}_{i, j}$ and $\overline{\mathcal {C}}_{i, j}$ as $\underline{\tau }_{i, j}^{1} \,<\,\cdots \,<\, \underline{\tau }_{i, j}^{C_{i, j}}$ and $\overline{\tau }_{i, j}^{1} \,<\,\cdots \,<\, \overline{\tau }_{i, j}^{C_{i, j}}$, respectively. Then dominance durations with respect to the *i*-th panelist correspond to $\overline{\tau }_{i, j}^{1} - \underline{\tau }_{i, j}^{1}$, $\ldots$, $\overline{\tau }_{i, j}^{C_{i, j}} - \underline{\tau }_{i, j}^{C_{i, j}}$. We can see that each value of $\overline{\tau }_{i, j}^{c} - \underline{\tau }_{i, j}^{c}$ corresponds to $Y_{i, j, c}$ defined in “Statistical methods”.

Additionally, the transition probability $P_{j, j'}$ is also defined as a SMC, but the definition is different from that for a traditional MC: $P_{j, j'}$ for $j \ne j'$ is calculated as $m_{j, j'} / \tilde{m}_{j, \cdot }$, where $\tilde{m}_{j, \cdot }$ is the total number of transitions from the *j*-th attribute to any attribute except itself, and the self-transition probability is defined as $P_{j, j} = 0$ for each *j*. Let $\varvec{Z}_{i}$ $=$ $\left( Z_{i}(1) \,,\,\ldots \,,\, Z_{i}(T_{i}) \right)$ be a vector consisting of sequences obtained from the *i*-th panelist.

The likelihood of a SMC given the sequences of all panelists, $\varvec{Z}_{1} \,,\,\ldots \,,\, \varvec{Z}_{I}$, is calculated by using $f_{i, j}$ in Eq. (1) as follows:

$$\begin{aligned} L(\varvec{Z}_{1} \,,\,\ldots \,,\, \varvec{Z}_{I}) \,=\, & \prod _{i=1}^{I} \, \delta _{Z_{i}(1)} \, \prod _{j=1}^{J} \, \left\{ \prod _{c \in \mathcal {C}_{i, j}^{\star }} P_{Z_{i}(\underline{\tau }_{i, j}^{c}-1), Z_{i}(\underline{\tau }_{i, j}^{c})} \right. \\ & \left. \quad \prod _{c=1}^{C_{i, j}} f_{i, Z_{i}(\underline{\tau }_{i, j}^{c})}(\overline{\tau }_{i, j}^{c} - \underline{\tau }_{i, j}^{c}) \right\} \,, \end{aligned}$$

where $\mathcal {C}_{i, j}^{\star }$ is given by

$$\begin{aligned} \mathcal {C}_{i, j}^{\star } \,=\, \Bigl \{ c \,\in \, \left\{ 1 \,,\,\ldots \,,\, C_{i, j} \right\} \;{\Big |}\; \overline{\tau }_{i, j}^{c} \,\ne \, T_{i} + 1 \Bigr \} \,, \end{aligned}$$

for $i \in \{ 1 \,,\,\ldots \,,\, I \}$ and $j \in \{ 1 \,,\,\ldots \,,\, J \}$. SMC can cope with a wide class of probability distributions other than GD, such as PD and NBD. The probability functions of GD, PD, and NBD are

3 $$\begin{aligned} f_{i, j}^{\mathrm {GD}}(y)= & {} \left( 1 - \rho \right) ^{y} \, \rho \,, \nonumber \\ f_{i, j}^{\mathrm {PD}}(y)= & {} \frac{\lambda ^{y}}{\Gamma (y+1)} \, \mathrm {e}^{-\lambda } \,, \nonumber \\ f_{i, j}^{\mathrm {NBD}}(y)= & {} \frac{\Gamma (y+\kappa )}{\Gamma (y+1) \, \Gamma (\kappa )} \, \left( \frac{\mu }{\mu + \kappa } \right) ^{y} \, \left( \frac{\kappa }{\mu + \kappa } \right) ^{\kappa } \,, \end{aligned}$$

respectively, where $\rho \in (0 ,\, 1)$, $\lambda > 0$, $\kappa > 0$, and $\mu \in \mathbf {R}^{1}$ are parameters in the corresponding probability distributions (see also Fig. S2 in Online Resource). The proposed regression models are expanded cases of NBD (see Eqs. (2) and (3)). The expectations of GD, PD, and NBD are $(1 - \rho ) / \rho$, $\lambda$, and $\mu$, respectively. $\kappa$ in NBD shapes the distribution without changing the expectation ($\mu$), and so is referred to as the shape parameter.

### A.3 Relationship among models

$Y \sim \mathrm {NBR}\left( \mu \,,\, \kappa \right)$ means that *Y* has a NBD with expectation $\mu$ and variance $\mu (\mu + \kappa ) / \kappa$. It can be proved that *Y* has a GD with $\rho = 1/(\mu +1)$ if $\kappa = 1$, and converges to a PD with $\lambda = \mu$ if $\kappa \rightarrow +\infty$. Hence, it is regarded that NBR models include regression models built upon GD and PD. Additionally, we can also prove that the model based on a SMC (e.g., Lecuelle et al. 2018) is actually a special case of a NBR model. If we employ none of the explanatory variables, the regression coefficient parameters of each attribute group $\varvec{\beta }_{1} \,,\,\ldots \,,\, \varvec{\beta }_{G}$ reduce to only the intercept term. In such a case, the expectation is expressed by a single parameter (the intercept term), and thus the model coincides practically with the basic NBD.

## Abbreviations

AIC: This is Akaike information criterion
GD: This is Geometric distribution
GLM: This is Generalized linear model
MC: This is Markov chain
NBD: This is Negative binomial distribution
NBR: This is Negative binomial regression
PD: This is Poisson distribution
SMC: This is Semi-Markov chain
TDS: This is Temporal dominance of sensations

## Code availability

The code used in this manuscript is not publicly available due to business reasons, but is available from the corresponding author on reasonable request.
