# Correction to: Statistical modeling for temporal dominance of sensations data incorporating individual characteristics of panelists: an application to data of milk chocolate

**DOI:** 10.1007/s13197-021-05311-1

**Published:** 2021-11-30

**Authors:** Sumito Kurata, Reiko Kuroda, Fumiyasu Komaki

**Affiliations:** 1 Graduate School of Information Science and Technology, The University of Tokyo, 7-3-1 Hongo, Bunkyo-ku, Tokyo, 113-8656 Japan; 2 LOTTE Co., Ltd. Central Laboratory, 1-1 Numakage 3-chome, Minami-ku, Saitama, 336-8601 Japan; 3 RIKEN Center for Brain Science, 2-1 Hirosawa, Wako City, Saitama, 351-0198 Japan

## Correction to: J Food Sci Technol 10.1007/s13197-021-05260-9

The article ‘‘Statistical modeling for temporal dominance of sensations data incorporating individual characteristics of panelists: an application to data of milk chocolate’’, written by Sumito Kurata, Reiko Kuroda and Fumiyasu Komaki was originally published electronically on the publisher’s internet portal on 24^th^ September, 2021 without open access. With the author(s)’ decision to opt for Open Choice the copyright of the article changed on 4^th^ October,2021 to © The Author(s) 2021 and the article is forthwith distributed under the terms of the Creative Commons Attribution 4.0 International License, which permits use, sharing, adaptation, distribution and reproduction in any medium or format, as long as you give appropriate credit to the original author(s) and the source, provide a link to the Creative Commons licence, and indicate if changes were made. The images or other third party material in this article are included in the article’s Creative Commons licence, unless indicated otherwise in a credit line to the material. If material is not included in the article’s Creative Commons licence and your intended use is not permitted by statutory regulation or exceeds the permitted use, you will need to obtain permission directly from the copyright holder. To view a copy of this licence, visit http://creativecommons.org/licenses/by/4.0/.

The original article has been updated.

