# Supplementary material for: Statistical modeling for temporal dominance of sensations data incorporating individual characteristics of panelists: an application to data of milk chocolate
Source: J Food Sci Technol. 2021 Sep 24;59(6):2420–8. doi: 10.1007/s13197-021-05260-9 (PMC9114240; doi:10.1007/s13197-021-05260-9)
Supplement: Supplementary file 1 — Supplementary file1 (PDF 413 kb) [file 13197_2021_5260_MOESM1_ESM.pdf]

Additional figures

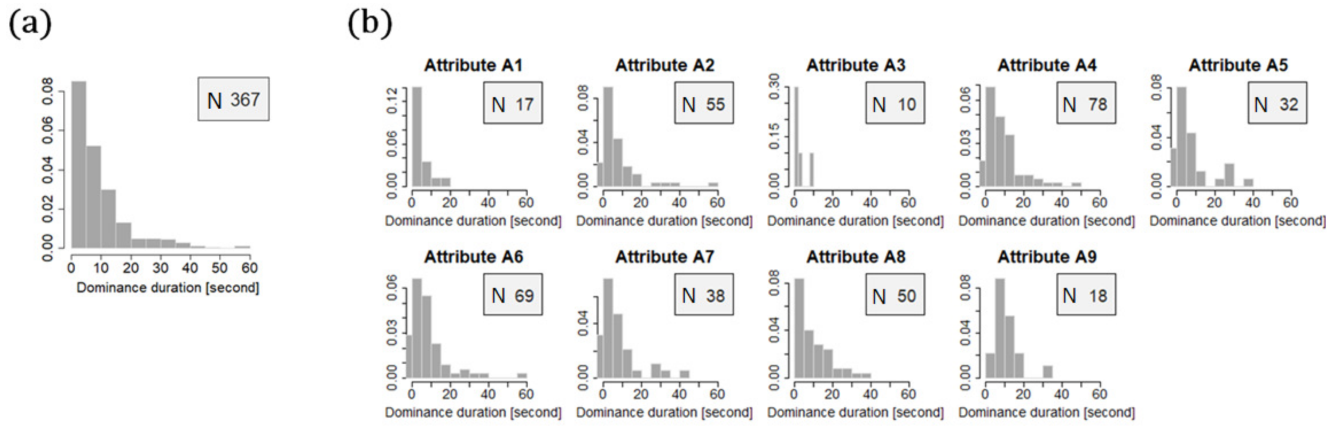

Fig. S1 [Related to the subsection "Sensory evaluation"]
(a) Histogram of dominance durations of TDS data obtained in our experiment. N indicates the total number of observed dominance durations; (b) Histograms of dominance durations divided according to attributes. N indicates the number of observed durations of the corresponding attribute being dominant

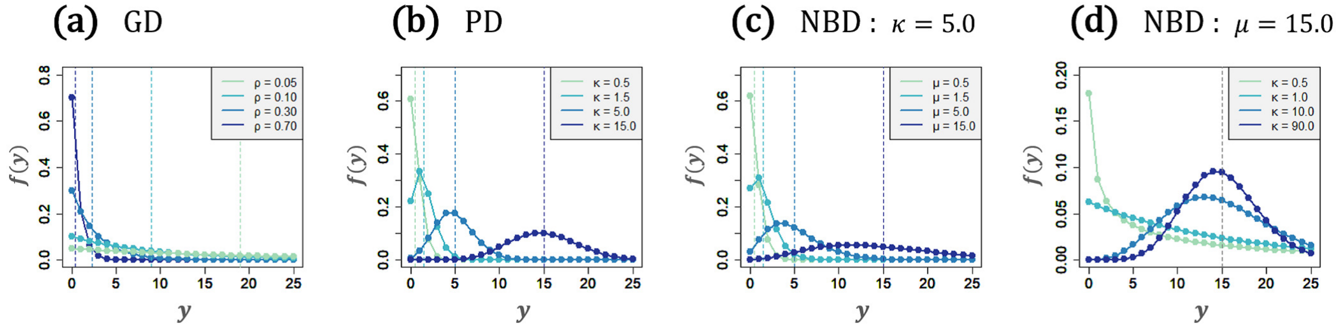

Fig. S2 [Related to "Semi-Markov chain" in the appendix]
Probability functions $f(y)$ of (a) GD, (b) PD, and (c, d) NBD for some values of parameters. The vertical dotted line is the expectation of the distribution with the corresponding parameter value. In (c) and (d), the values of shape parameter (κ) and expectation parameter (μ) are fixed as 5.0 and 15.0, respectively

Estimated results of real data analysis by conventional models [Related to the section "Results and Discussion"]

Tables **S1**, **S2**, and **S3** show estimates of the parameters in each of Model G, Model N, and Model Ng, respectively. From "Results and Discussion", we find that a SMC with NBD (Model N) is more suitable than a GD (Model G) for modeling dominance durations. Actually, the values of the shape parameters in the NBD models, denoted by κ in Tables **S2** and **S3**, are not close to 1 (NBD coincides with the GD with $\rho = 1/(\mu + 1)$ if $\kappa = 1$). By dividing the values of the parameters according to attribute groups, we can express the differences of tendencies between the attributes flexibly (Model Ng).

Table S1 Estimated value of parameter (ρ) in Model G

Attribute	ρ
All	0.10

Table S2 Estimated values of expectation parameter (μ) and shape parameter (κ) in Model N

Attribute	μ	κ
All	10.37	1.86

Table S3 Estimated values of expectation parameter (μ) and shape parameter (κ) of each attribute group in Model Ng

Attribute group	μ	κ
I	6.37	3.76
II	10.37	1.73
III	11.07	1.91
IV	12.94	6.78
